# Sexual Attitudes, Beliefs, Practices, and HIV Risk During Pregnancy and Post-delivery: A Qualitative Study in Malawi, South Africa, Uganda, and Zimbabwe

**DOI:** 10.1007/s10461-021-03454-y

**Published:** 2021-12-14

**Authors:** Julia H. Ryan, Alinda Young, Petina Musara, Krishnaveni Reddy, Nicole Macagna, Victor Guma, Linly Seyama, Jeanna Piper, Ariane van der Straten, the MTN- 041/MAMMA Study Team

**Affiliations:** 1 Women’s Global Health Imperative (WGHI), RTI International, Berkeley, CA USA; 2 University of Zimbabwe College of Health Sciences Clinical Trials Research Centre, Harare, Zimbabwe; 3 Wits Reproductive Health and HIV Institute (Wits RHI), University of the Witwatersrand, Johannesburg, South Africa; 4 FHI 360, Durham, NC USA; 5 Makerere University – Johns Hopkins University Research Collaboration, Kampala, Uganda; 6 Johns Hopkins Project-College of Medicine, University of Malawi, Blantyre, Malawi; 7 DAIDS, NIH/NIAID, Bethesda, MD USA; 8 Center for AIDS Prevention Studies (CAPS), University of California San Francisco, San Francisco, CA USA

**Keywords:** HIV prevention, Sexual behavior, Sub-Saharan Africa, Pregnancy, Postpartum

## Abstract

Women who acquire HIV during the pregnancy and breastfeeding periods have a higher risk of transmitting the virus to their child than women who become infected with HIV before pregnancy. We explore the context of sexual beliefs and practices that may shape both HIV risk and willingness to use HIV prevention products during pregnancy and postpartum in Malawi, South Africa, Uganda and Zimbabwe. Twenty-three single sex focus group discussions and 36 in-depth interviews took place between May and November 2018 with recently pregnant or breastfeeding women, men, mothers and mothers-in-law of pregnant or breastfeeding women, and key informants. Participants across study groups and sites (N = 232) reported various perceived benefits and harms of sex during pregnancy and postpartum. Participants discussed reasons why men might seek sex outside of the relationship. There is a critical need for alternative prevention options to protect pregnant and breastfeeding women from HIV.

## Background

Fertility rates are high in sub-Saharan Africa, where women commonly spend a substantial portion of their reproductive years either pregnant or breastfeeding [1]. In addition, women and girls account for 58 percent of all new adult HIV infections in sub-Saharan Africa [2]. Women who acquire HIV during the pregnancy and breastfeeding periods have a higher risk of transmitting the virus to their infant than women who become infected with HIV before pregnancy [1]. Findings from existing literature suggest that women’s risk of acquiring HIV is heightened during the pregnant and lactating periods due to social and behavioural factors in addition to biological ones [3, 4]. In sub-Saharan Africa as elsewhere, sexual activity decreases as pregnancy progresses in most couples [5, 6] and often ceases completely during the lactating period [7], which may lead male partners to seek other sexual partners during this time [7, 8].

Condoms are used as both a contraceptive and HIV prevention method. However, condoms are not used with regularity during pregnancy or postpartum [1, 3, 5, 8]. Studies also report that men may discourage condom use due to a belief that sperm is beneficial for the growth and development of the fetus [9]. Regular and repeat testing allows for early identification of incident HIV infections and provides opportunities for early treatment access when necessary [10]. Due to the absence of repeat HIV testing, HIV seroconversions that occur late in pregnancy or during the postpartum period may go undetected [1], resulting in an increased risk of perinatal HIV transmission.

Evidence suggests that there are different cultural beliefs about having sex during pregnancy and postpartum, but questions remain about the extent to which key stakeholder perspectives, including male partners and other influencers, shape sexual attitudes and practices during these periods. In some sub-Saharan African countries, beliefs around sexual practices during the pregnancy and breastfeeding periods are passed down by female elders to new mothers [5]. These elders typically advise sexual abstinence in the last trimester of pregnancy, and resumption at six months postpartum [5]. Postpartum abstinence is often justified by the belief that the sperm will poison a mother’s breastmilk, thereby harming the nursing infant [7, 11]. In certain ethnic groups, family members such as mothers, sisters, and mothers-in-law come to stay with the breastfeeding mother, not only to help with household chores but also ensure that the couple observes the period of abstinence [11]. Despite advice to the contrary by elders in their community, some women continue to engage in sex during pregnancy with the belief that intercourse will widen their birth canal and facilitate an easy delivery, improve fetal well-being, and also maintain relationship harmony and prevent spousal infidelity [4, 9, 12–15].

### Parent Study

The MTN-041/MAMMA (Microbicide/PrEP Acceptability among Mothers and Male Partners in Africa) study was a multisite qualitative study with a total of 232 participants. Previous publications focused on perspectives about whether two new HIV prevention products, oral PrEP and a microbicidal vaginal ring would be accepted by pregnant/breastfeeding women and key influencers [16, 19]. Here, we explore sexual beliefs and practices that may shape both HIV risk and willingness to use HIV prevention products during pregnancy and postpartum in these four sub-Saharan African settings. This analysis allows for examination of these questions across four distinct locations and the opportunity to identify consistent findings as well as distinct factors. The paper augments existing literature on the perceived benefits and downsides of sex during pregnancy and postpartum by highlighting perspectives of women and key groups influential to these decisions.

## Methods

### Study Design

The MTN-041/MAMMA study was conducted in Blantyre, Malawi; Johannesburg, South Africa; Kampala, Uganda; and Chitungwiza, Zimbabwe. This parent study explored perceptions of HIV risk and attitudes about the use of a monthly vaginal ring and daily oral pill during pregnancy and breastfeeding in preparation for two phase 3b prevention trials in pregnant and breastfeeding women. Participants enrolled between May and November 2018. Twenty-three single sex Focus group discussions (FGDs) took place across the four sites with participants who were independently recruited into the following groups: (1) HIV-uninfected women aged 18–40 who were currently or recently (in the past two years) pregnant or breastfeeding (referred to as women); (2) men aged 18+ with female partners who were currently or recently pregnant or breastfeeding (referred to as men); (3) mothers or mothers-in-law of women who were currently or recently pregnant or breastfeeding (referred to as grandmothers). All FGDs consisted of between 5 and 12 participants. Additionally, In-depth interviews (IDIs) were conducted with 36 key informants, including clinicians, traditional care providers, social service/community health workers, and community leaders. Six key informant interviews were conducted in Johannesburg, and ten in each of the other three sites.

### Data Collection

FGDs and IDIs were conducted by trained social scientists in English or local languages, which included Zulu, Luganda, Chichewa, and Shona using semi-structured guides. Topics explored were the same across study groups and included sexual activity, sexual health practices, health-related decision making, HIV risk perception, and opinions about various HIV prevention methods. FGDs and IDIs were audio recorded, transcribed and translated to English when necessary.

Demographic information was collected for all participants through interviewer-administered questionnaires. Women and men also completed an interviewer-administered behavioural assessment prior to the FGD that focused on sexual behaviour and pregnancy and breastfeeding history. Pseudonyms are used to protect the identity of participants.

### Data Analysis

A codebook was iteratively developed as previously described, and transcripts were coded using Dedoose software v7.0.23 by a team of five analysts [19, 20]. This was a secondary analysis in which data coded with the code SEX, defined as *anything about sex practices or behaviors including frequency, position, libido, sperm, pleasure, and abstinence*, were extracted. Data were stratified by participant type (i.e., women, men, grandmothers, and key informants) and study site and were analyzed thematically by two analysts (JR and AY), who extracted relevant data into a matrix that included illustrative quotations. Analysts held biweekly meetings to review emerging themes, similarities, or differences across participant types and study sites. Consensus decisions and action items were documented systematically with notes.

### Research Ethics

The MTN-041 protocol was approved by Western Institutional Review Board (WIRB), an independent IRB located in Olympia, Washington, USA, and by local IRBs at each of the study sites. The study was overseen by the regulatory infrastructure of the US National Institutes of Health and the Microbicide Trails Network. All participants provided written informed consent prior to participation.

## Results

We explored prominent themes related to attitudes, beliefs, and practices around sex during pregnancy and postpartum that emerged from questionnaires and qualitative data (FGDs and IDIs) with the four stakeholder groups included in the multi-country study.

Basic demographics of the study sample are presented in Tables 1 and 2. Across study sites, 78% of women reported living with their spouse or primary sex partner. Most women and men reported having vaginal sex with their primary partner/spouse in the past three months. During the pregnancy and breastfeeding periods, a majority of women and men agreed that decisions around having sex were made jointly between partners (Table 1). Despite this agreement, our qualitative data suggest that women and men may have different intentions behind their decisions when it comes to having sex. For example, women often agreed to have sex to prevent their partner from finding outside sexual partners, whereas men more commonly desired sex for their own satisfaction. These differences are explored in more depth below.

**Table 1:** Demographic and baseline characteristics of women (N = 65) and men (N = 63)

Variable	Women N = 65	Men N = 63
Location (site)		
Blantyre	15	16
Kampala	18	19
Johannesburg	15	12
Chitungwiza	17	16
Median age (range)	26 (19–40)	30 (19–54)
Secondary education completed	33 (51%)	35 (56%)
Married or living with partner	50 (77%)	51 (81%)
Currently pregnant/partner currently pregnant	32 (50%)	20 (32%)
Vaginal sex with primary partner in last 3 months	55 (87%)	53 (88%)
Awareness of male condoms	65 (100%)	63 (100%)
Ever use male condoms	51 (79%)	60 (95%)
Primary partner at time of FGD		
Yes	63 (97%)	60 (95%)
No	2 (3%)	3 (5%)
Median # years with current spouse/primary partner (range)	5 (1–21)	4 (0–29)
[Women] Mean number of pregnancies resulting in live birth (range)	2 (0–6)	N/A
[Men] Mean number of children participant has fathered (range)	N/A	2 (0–8)
[Women] Spouse/primary partner is father of baby		
Yes	60 (92%)	N/A
No	3 (5%)	N/A
No primary partner	2 (3%)	N/A
[Women] Ever breastfed*	48 (74%)	N/A
[Women] Where you received care during recent pregnancy		
Doctor	23 (35%)	N/A
Nurse	52 (80%)	N/A
Traditional birth attendant	2 (3%)	N/A
Other traditional healer	2 (3%)	N/A
Other	6 (9%)	N/A
During pregnancy, who has more say when making decisions about having sex		
You	10 (15%)	13 (21%)
Him/her	18 (28%)	10 (16%)
Both equally	35 (54%)	40 (64%)
Not applicable	2 (3%)	0
During breastfeeding, who has more say when making decisions about having sex		
You	4 (8%)	10 (16%)
Him/her	10 (21%)	9 (14%)
Both equally	32 (67%)	36 (57%)
Not applicable	2 (4%)	8 (13%)

*Includes nulliparous women who were pregnant for the first time

*N/A* not asked

**Table 2:** Demographic characteristics of key informants (N = 36) and grandmothers (N = 68)

Variable	Key informants N = 36	Grandmothers N = 68
Location (site)		
Blantyre	10	10
Kampala	10	21
Johannesburg	6	20
Chitungwiza	10	17
Sex		
Male	11 (31%)	0
Female	25 (69%)	68 (100%)
Role in community		
Nurse	5 (14%)	N/A
Religious leader	7 (19%)	N/A
Social service provider	3 (8%)	N/A
Traditional care provider	7 (19%)	N/A
Clinical doctor	3 (8%)	N/A
Community health worker	2 (6%)	N/A
Mean age (range)	50 (25–79)	50 (36–69)
Secondary education completed	28 (78%)	19 (28%)

*N/A* not asked

**Fig. 1:**
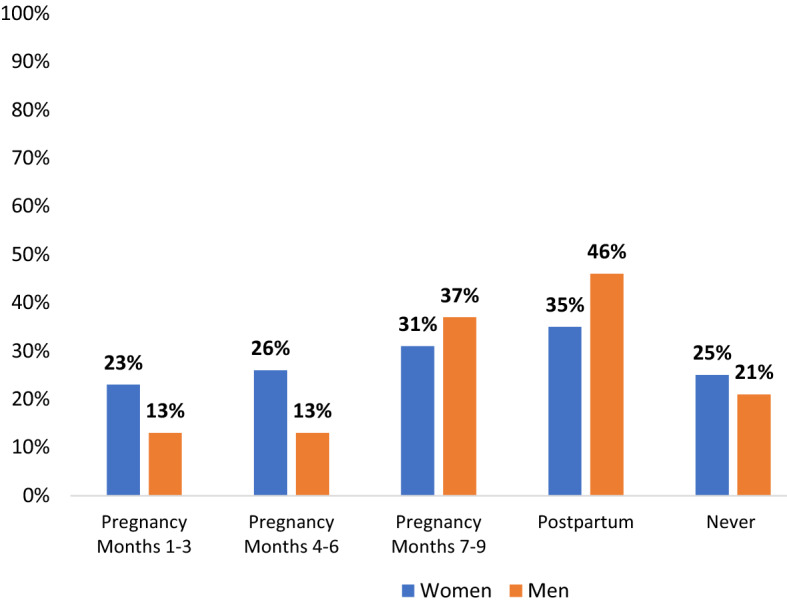
Periods of abstinence during recent pregnancy and postpartum for women (N = 65) and men (N = 63)

Women and men also reported abstinence during pregnancy or post-partum periods (Fig. 1): overall, 75% of the women reported practicing abstinence at some point(s) during pregnancy or postpartum. However, there was no given period in which more than 35% of women reported abstinence, indicating that the timing of abstinence differed between participants. Abstinence increased in frequency as the pregnancy progressed (from 23 to 31%), and women most frequently reported postpartum abstinence (35%). Similarly, 79% of men reported practicing abstinence with their primary partner/spouse, with highest rates of abstinence occurring postpartum (44%) and in the final trimester of her pregnancy (37%).

### Beliefs, Attitudes, and Practices About Sex During Pregnancy and Postpartum

During FGDs and IDIs, participants shared diverse views about the cultural beliefs that impact sexual behaviour during the pregnancy and postpartum periods. While the findings presented here reflected variation on individual, study group, and site levels, there was more consistency expressed across groups and sites than there were differences.

#### Pregnancy: Benefits of Sex

Across all sites, participants mentioned that sperm is slippery and helps to open the birth canal, thus facilitating delivery. By this logic, participants explained that having sex close to the time of delivery is healthy and encouraged by health care providers (HCPs). Overall, participants also mentioned that sperm is seen as nutritious and contributing to the growth and development of the fetus:

*Some say when you are pregnant you must have sex so that the baby can grow, the sperms grow the baby.* (Red, pregnant/breastfeeding woman, age 30, Johannesburg)

Men in Malawi and South Africa felt that unprotected sex is beneficial during pregnancy because of the belief that the sound that sperm makes when it hits the womb energizes the fetus. This also means that a condom would interfere with the process. Enoch explains the importance of fetal exposure to sperm:

*...Basically when they say you get a healthy baby they mean your baby becomes energetic when you don’t use condoms, and when you ejaculate inside your woman apparently the sperms hit the womb and because of that sound the foetus become energetic because when the sperms get there the foetus becomes lively.* (Enoch, man, age 33, Johannesburg)*.*

The perceived positive effect of unprotected sex that Enoch and others explained disincentivizes men from using condoms with their pregnant partners and may contribute to women’s risk of acquiring HIV during this time.

#### Pregnancy: Harms of Sex

A dominant theme among women was a concern that having sex close to delivery results in the baby coming out covered in sperm. One woman in Zimbabwe explained that this is not only deeply embarrassing for the mother, but is also seen as unhygienic:

*As we grew up we were taught that after 7 months, you are not supposed to have sex with your husband because those sperms will be all over the baby such that you can even hear them saying, “Your baby is disgusting, your baby is coming out covered like this, and you are untidy.”* (Tendai, pregnant/breastfeeding woman, age 37, Chitungwiza)

This view was echoed widely by women across sites who attributed sex in close proximity to delivery to various adverse effects for the baby. Further, at all sites but Uganda, participants stated that sex at certain stages of pregnancy or in certain positions will potentially hurt the baby’s head. Several women and grandmothers pointed to the balancing act of negotiating this risk to the baby with a male partner’s sexual satisfaction. One grandmother in Chitungwiza explains that while sex is no longer prohibited late in pregnancy, there are precautions that should be taken:

*But things are now changing. Sex is no longer prohibited, you can have it anytime. But you should be careful on how you do it. You should avoid very deep penetrations so that you do not hurt the baby’s head. You just do it to make him [man/partner] feel satisfied*. (Mbuya Rutendo, grandmother, age 44, Chitungwiza)

#### Postpartum: Benefits of Sex

To maintain harmony in the spousal relationship, participants explained that having sex throughout the pregnancy and postpartum periods may be beneficial in that it keeps both partners sexually satisfied and prevents either one from seeking sexual partners outside of the relationship, a practice more commonly described for men. Thus, continued spousal sexual relations were perceived to reduce the risk of introducing HIV into the relationship. This was a popular view in all study groups. In their interviews, HCPs noted that they typically encourage women to have sex throughout their pregnancy until they go into labor, and to resume sex about 6 weeks after delivery in order to allow sufficient time for healing and recovery.

#### Postpartum: Harms of Sex

Participants at all sites highlighted community views that sex and sperm may have detrimental effects for the baby or for the parents after delivery. Having sex while the baby is “too small,” for example, was widely considered dangerous for the baby’s growth and development. In Zimbabwe, grandmothers mentioned that frequent sex during the breastfeeding period may cause the baby to lose weight:

*When we were growing up we were told that when you are breastfeeding…you should not frequently have sex because the baby will lose weight.* (Mbuya Tsitsi, grandmother, age 42, Chitungwiza)

Participants across sites echoed the belief that when resumed “too early” after delivery, sex causes the baby to lose weight and become weak. Men and grandmothers in Malawi described a specific illness called *Tsempho* that presents with weight loss and weakness, either for the baby or the husband, and occurs when sex is resumed too soon after delivery:

*Let me just say more on that, they say that if a woman have sex with her husband before the end of the six-months period…the child suffers from ‘Tsempho’…* (Mike, man, age 21, Blantyre)

Despite wanting to preserve their sexual relationships, it was widely acknowledged across groups that women often do not want to have sex at certain times during the pregnancy and post-delivery periods due to physical discomfort, pain, healing (postpartum), hormonal changes, or general sexual disinterest. Regardless of women’s desires and preferences, discussions around sex frequently emphasized the goal of satisfying men’s needs to both preserve the relationship and mitigate the risk of HIV acquisition, if he seeks concurrent sexual relationships.

*…If you ask breastfeeding mothers what their motive for sex is, they will tell you that they are just having sex to satisfy their husbands so that they don’t venture into other sexual relationships.* (Key informant (family planning counselor), man, age 50, Chitungwiza)

Women across sites explained that consideration for a woman’s personal health and healing process from delivery factors into decision making about appropriate timing to resume sex:

*It may also depend on the type of delivery the woman had. Others deliver through operation and others normal but they do have vaginal tears. So, it all depends on the type of delivery that will ensure you do not feel pain once you resume sex.* (Lucy, pregnant/breastfeeding woman, age 29, Blantyre)

### HIV Risk Behaviours During Pregnancy and Postpartum

While a majority of women and men agreed that having sex during pregnancy and postpartum is a joint decision between partners, underlying reasons for having sex may stem from various beliefs regarding partners’ needs and desires for themselves and their families.

Overall, participants reported that men find other sexual partners during these maternal periods for many reasons, including women’s disinterest in sex, men’s lack of attraction to their partner, culturally prescribed periods of abstinence, fear of hurting the baby (particularly during later stages of pregnancy), and fear of conceiving during the postpartum period. Men themselves spoke openly about their beliefs and tendencies to find sex outside of their relationship during these maternal stages. They often described a tension between their perceived biological drive for sex and “sparing” their pregnant partner from sex she (presumably) did not want to have. Despite this tension, men explained that they are expected to have sex with their wives:

*Most men believe that when women are pregnant, they must go out, and reserve their wives [Spare their wives from sex]. So that is where the challenge is because that is when you get whatever you get there [Acquire HIV]. You come back home and want to fulfil your conjugal duties….In doing so, you will be infecting her with the disease that you would have acquired from outside*. (Pizza, man, age 28, Chitungwiza)

In addition to recognizing the risk men introduce into their relationships, participants across sites and study groups explained that when left sexually unsatisfied, women may also seek other sex partners during the pregnancy and postpartum periods. Of note, this concern was voiced by both women and men. Men highlighted that this is something husbands have a responsibility to prevent by keeping their partners happy and sexually satisfied.

*If left unsatisfied, then the problem will come that she will invite another man into the house [for sex]. She has nothing to fear in terms of getting pregnant because she is already pregnant of you [husband]. So this invited man may bring the HIV...A loving man must love his wife and make her happy when having sex.* (Boloma, man, age 53, Blantyre)

Like Boloma, other participants stated that couples where the woman is pregnant are less motivated to use a condom when preventing pregnancy is not necessary, thus introducing another layer of HIV risk.

*And when you are sleeping [having sex] with your partner while pregnant the partner would be like there is no need for a condom because you are already pregnant so let’s just not use it at all, while you don’t know what he is doing when you are not there so it’s very high risk to get infected while pregnant.* (Apple, pregnant/breastfeeding woman, age 24, Johannesburg)

Women at several sites echoed this belief that pregnant women and their partners are less likely to use condoms than couples who are trying to prevent pregnancy.

Participants expressed varying views of a woman’s attractiveness during pregnancy and linked these perceptions to an increased risk for HIV during pregnancy. There was a lack of consensus: many participants believed that men find pregnant women more attractive, which puts them at risk because they are highly sought after by other men. These divergent perceptions are expressed by three different participants below.

*Men believe that if a woman is pregnant they have hot bodies and they enjoy sex with pregnant women more than with women who are not pregnant. So, if she is a pregnant single mother they would want sex with her, by so doing she gets exposed.* (Key informant (religious leader), woman, age 51, Kampala)

A South African grandmother echoed a common perspective across study groups in explaining that a pregnant woman is at high risk for HIV not only because she is seen as sexy and desirable by men, but also because she wants sex from men during this time:

*Pregnant women become so beautiful and you would hear a man saying, “Yes if my wife could be pregnant,” so they are at high risk because it means some internal things attract men…So you might get it [HIV] by accident because a woman is sexy she would find herself sleeping with a wrong person. So the truth is pregnant women becomes attractive and they also want the men.* (Guest, grandmother, age 67, Johannesburg)

In contrast to this belief, some male participants explained that women are viewed as *unattractive* during pregnancy, leading men to crave sex with other women than their spouse:

*And the man himself looks at the pregnant [woman] and sees that she is no longer attractive. Then you end up getting some other woman. And when the woman is pregnant, she can easily contract diseases especially those that are sexual.* (Joy, man, age 28, Kampala)

Thus, discussion related to attractiveness during pregnancy was framed in the context of heightened HIV risk, both for participants who believed women are more attractive (e.g., they are pursued by men for sex) and those who thought women are less attractive during pregnancy (e.g., their partners seek sex outside of the relationship).

In discussions about the above HIV risk behaviours, women and men described several HIV prevention strategies including HIV testing, condom use, abstinence and being faithful to their partner. There were frequent references to men rejecting condom use and HIV testing because of a fear of knowing their HIV status as well as a reluctance to acknowledge their own risk behaviours. Barbra explains that one way men avoid HIV testing is by choosing to adopt their partner’s negative test result as their own—a particularly risky assumption during pregnancy:

*One of the challenges we find is that men usually decline to go for HIV testing…They usually say “You can go for testing, if they find you HIV negative then I am also HIV negative.” That is one of the biggest challenges we face; men fear to be tested for HIV yet they are promiscuous.* (Barbra, pregnant/breastfeeding woman, age 36, Kampala)

## Discussion

This analysis explored beliefs, attitudes, and sexual practices during the pregnancy and postpartum periods in sub-Saharan Africa, and how these beliefs may inform women’s potential HIV risk during those periods of life. While many of the findings build on existing work, this study was notable in that it incorporated a diverse range of voices from understudied populations. A diverse sample of stakeholders from four countries was included, covering a range of roles, ages, languages, educational backgrounds, and family/living situations. Both women and men reported engaging in less frequent sexual activity as pregnancy progressed, and also observing postpartum abstinence. Study groups voiced a number of conflicting beliefs related to sexual behaviour during pregnancy and postpartum. For example, there was a widely held belief that having sex throughout pregnancy helps to prepare the birth canal and leads to an easy delivery. At the same time, participants in all groups cited fears of adverse effects resulting from sex, often highlighting concerns about harming the baby later in pregnancy. Participants also discussed the benefits of sexual practices during pregnancy or postpartum for satisfying both partners, but especially men, and thus preventing infidelity and reducing HIV risk. These findings build on several other global studies that show changes in the frequency of sexual activity during pregnancy due to cultural traditions and beliefs [17, 18].

These attitudes and beliefs concerning sex influenced the guidance and advice from key influencers to pregnant and breastfeeding women. For example, men in Malawi described an illness called *Tsempho* that affects a newborn child when the parents do not abstain from sex for at least six months after delivery [19, 20]. Based on this belief, these men voiced opinions that partners should be sexually abstinent until 6 months post-delivery to prevent adverse effects for the baby. Other participants diverged in their opinions on the appropriate timing to resume sexual practices after delivery, spanning from a few weeks to many months. Many participants recognized that this decision depends largely on a woman’s individual path to recovery following her delivery. Nevertheless, the potential risk of introducing HIV into the relationship via spousal infidelity contributed to a common belief that shorter periods of prenatal and postpartum abstinence are preferable.

Participants across study groups and sites reported reasons why each partner might seek sex outside of the relationship, including a lack of sexual attraction, a desire to spare the pregnant woman from discomfort or prevent harm to the fetus, and a respect for culturally prescribed periods of abstinence. We previously reported widespread recognition that pregnant and breastfeeding women are at high risk for HIV, primarily due to the sexual behaviour of their male partners, and that prevention options other than condoms are critical for this population of women [16]. All groups and sites, including men themselves, spoke about men as the primary source of HIV risk for pregnant and breastfeeding women. However, other risk factors are also highlighted here, including a lack of consistent use of prevention methods and the perception that pregnant women are more attractive and may have an increased desire for sex compared with their non-pregnant counterparts. Moreover, condom use is especially low among pregnant couples who associate condoms with contraception, since becoming pregnant is not a concern. Previous studies also highlight less frequent condom use during pregnancy and postpartum, corroborating our findings [5, 14].

Contradictory beliefs and traditions came out clearly in this study, even among the women themselves, who acknowledged that their sexual needs and desires evolve over time due to the physiological changes in their bodies and information they receive from key influencers in their lives. Our data suggest that pregnant and breastfeeding women receive advice and support related to their health decision-making and sexual behaviour from male partners, HCPs, mothers and mothers-in-law, sisters, aunts, and others who have unique and sometimes conflicting perspectives on the benefits and risks of certain behaviours. Participants recognized their differing perspectives related to the timing and implications of sexual practices during pregnancy and postpartum. In previous publications, we found that HCPs are overwhelmingly a trusted source of advice and guidance for women regarding HIV prevention product use [16, 21]. In this study we learned that HCPs are also a trusted source when it comes to decision making around sex during pregnancy and while breastfeeding. However, there are others, most notably a woman’s partner, whose perspectives are influential on matters related to sex and relationships. The extent to which women follow advice from various key influencers in their lives should be further investigated, and in cases where there are contradictory beliefs, questions remain about how women navigate making decisions related to sex [22].

This study had several limitations. A majority of women who participated in FGDs were recruited from antenatal and postnatal clinics in urban and peri-urban settings, highlighting a selection bias. The perspectives and experiences discussed in this paper do not include women without access to health care facilities who may be residing in more rural settings. Men and grandmothers were recruited from various community settings, including through street outreach, construction sites (men) and religious gatherings (grandmothers). In addition, the primary focus of the study was to explore attitudes and beliefs during the pregnancy period. This was reflected in the IDI guide, where all topics were first asked about pregnancy, and subsequently probed about the postpartum and breastfeeding periods, resulting in less detail and depth for data post-delivery. Nevertheless, because the study was conducted across a variety of countries with broad ethnic representation and multiple stakeholder groups, the study provides rich insight into regional perspectives on sexual behaviour during pregnancy and postpartum.

This study makes clear the limitations of currently available HIV prevention methods for pregnant and postpartum women and identifies cultural practices and beliefs that make HIV prevention difficult. Further studies could build upon these findings to identify prevention options that are better suited to a couple’s lifestyle, risk factors, and needs. The DELIVER and B-PROTECTED trials are ongoing and seek to evaluate the safety and pharmacokinetics of women’s use of oral PrEP or a microbicidal vaginal ring during pregnancy and breastfeeding and include assessment of facilitators and barriers to product adherence [23, 24].

## Conclusions

This study illuminates why pregnant and postpartum women in Malawi, South Africa, Uganda, and Zimbabwe are at high risk of contracting HIV in the context of limited prevention options. Partner infidelity during periods of marital abstinence coupled with condom non-use puts women and their children at increased risk of HIV. Addressing these cultural realities requires increased understanding of sexual beliefs and practices as well as alternative HIV prevention options, such as the vaginal ring, that are suitable for the unique realities of couples during pregnancy or postpartum.
